# Identification and functional validation of a novel disease‐causing variant in the noncoding region of *NYX*

**DOI:** 10.1111/aos.70094

**Published:** 2026-02-23

**Authors:** Filip Spanic, Christelle Michiels, Julien Navarro, Aline Antonio, Christel Condroyer, Camille Andrieu, Billy Gipsy, Sylvie Berthémy‐Pellet, Mathilde Gallice, Isabelle Audo, Christina Zeitz

**Affiliations:** ^1^ Sorbonne Université, INSERM, CNRS, Institut de la Vision Paris France; ^2^ Centre Hospitalier National d'Ophtalmologie des Quinze‐Vingts Centre de Référence Maladies Rares REFERET and INSERM‐DGOS CIC 1423 Paris France; ^3^ Centre de Référence Anomalie du développement Service de génétique CHUGA ‐ Hôpital Couple Enfant Grenoble France; ^4^ Department of Ophthalmology University of Grenoble Grenoble France

**Keywords:** cCSNB, high myopia, noncoding variant, NYX, splice‐site mutation

## Abstract

**Purpose:**

Inherited retinal diseases (IRDs) are a clinically and genetically heterogeneous group of disorders, with ~30% of cases remaining genetically unsolved. Complete congenital stationary night blindness (cCSNB) is a subtype of IRD, usually associated with reduced visual acuity, nystagmus and high myopia. Most cases are caused by variants in *NYX*, *TRPM1*, *GRM6*, *GPR179* or *LRIT3*. This study aimed to identify the genetic defect in a subject with clinically diagnosed cCSNB lacking coding variants in known associated genes.

**Methods:**

A male patient presented with an electroretinogram profile consistent with cCSNB in the absence of high myopia. Pathogenic variants were not detected using Sanger sequencing of the coding regions of all known CSNB‐associated genes. Whole genome sequencing (WGS) and bioinformatic analysis using SpliceAI, Pangolin, REVEL, CADD v1.7, BayesDel and MetaRNN were performed to detect potential pathogenic variants. Functional impact of this variant has been analysed using LINSIGHT, ReMM and FunUV. A minigene assay was used to assess the splicing impact of the identified variant.

**Results:**

WGS identified a novel c.‐57G>A variant in the 5′ untranslated region, within exon 1 of *NYX* coding for nyctalopin. In silico predictions suggested this variant to alter splicing, which was confirmed by a minigene assay showing abnormal expression of *NYX*. The defect was predicted to reduce nyctalopin production, potentially explaining the milder cCSNB phenotype.

**Conclusions:**

To our knowledge, this is the first report describing a noncoding variant in *NYX* causing CSNB but lacking high myopia. These results highlight the clinical importance of screening noncoding regions of known IRD genes in genetically unsolved cases. Whether the development of high myopia in cCSNB depends on the type and location of *NYX* variants remains to be elucidated.

## 1 INTRODUCTION

Inherited retinal diseases (IRDs) are a group of diseases characterized by their clinical and genetic variability (Berger et al., 2010; den Hollander et al., 2010; Sahel et al., 2015). With approximately 5.5 million people affected worldwide, IRDs cause vision loss due to the dysfunction or degeneration of the photoreceptors or the retinal pigment epithelium (Ben‐Yosef, 2022). In 50%–70% of IRD cases, the phenotype of affected subjects is caused by previously identified gene defects, with disease‐causing variants identified in more than 300 genes (Daiger et al., 2018; Retinal Information Network, 2025). Other patients exhibit the same IRD phenotype but lack variants in known genes, requiring detailed screening, combining sequencing methods with bioinformatics tools and functional analysis. In these subjects, the condition is caused by newly identified gene defects or by copy number variants, variants in noncoding or regulatory regions, which may also alter the splicing of the gene or the function of the protein (Carss et al., 2017; Jamshidi et al., 2019; Qian et al., 2021; van Cauwenbergh et al., 2017; Zeitz et al., 2013).

One of the forms of IRD is congenital stationary night blindness (CSNB) (Zeitz et al., 2015). This is a group of largely non‐progressive retinal disorders that primarily affect signal processing within photoreceptors, retinoid recycling in the retinal pigment epithelium (RPE) or signal transmission to retinal bipolar cells (BCs) (Zeitz, 2007; Zeitz et al., 2015; Huang et al., 2024). CSNB exhibits clinical and genetic heterogeneity, with inheritance patterns that can be X‐linked, autosomal recessive or autosomal dominant.

A major form of CSNB is complete CSNB (cCSNB), characterized by decreased visual acuity not only in dim light but also in daylight conditions, nystagmus, often strabismus and high myopia (MacDonald et al., 2008; Zeitz et al., 2015). It represents a signal transmission defect from photoreceptors to ON‐BCs with diagnosis aided by distinctive waveform changes in dark‐adapted (DA) and light‐adapted (LA) full‐field electroretinogram (ff‐ERG) (Miyake, 1986). Pathogenic variants in different genes including *NYX* [MIM# 300278], *GRM6* [MIM# 604096], *GPR179* [MIM# 614515], *TRPM1* [MIM# 603576] and *LRIT3* [MIM# 615004] lead to this condition in both affected individuals and mouse models (Audo et al., 2009, 2012; Bech‐Hansen et al., 2000; Dryja et al., 2005; Gregg et al., 2003; Koike et al., 2010; Li et al., 2009; Masu et al., 1995; Miyake, 1986; Morgans et al., 2009; Neuillé et al., 2014; Orhan et al., 2021; Peachey et al., 2012; Pusch et al., 2000; Shen et al., 2009; van Genderen et al., 2009; Zeitz et al., 2005, 2013). These genes code for proteins located in the outer plexiform layer of the retina and are crucial for signalling from photoreceptors to ON‐BCs (Zeitz et al., 2015). This localization is also reflected in the phenotype.

cCSNB is characterized by selective ON‐BC dysfunction. Typically, the ff‐ERG shows absent scotopic responses to a dim flash, leading to the term ‘complete CSNB’ (Zeitz et al., 2015). At low flash strength (DA 0.01), the b‐wave is absent. In response to a bright flash, only the b‐wave is reduced, while the a‐wave remains normal (DA 3 and DA 10), which is consistent with normal rod function and results in an electronegative ff‐ERG waveform (Miyake, 1989). The photopic responses are less affected: The LA 30 Hz ff‐ERG is often of normal amplitude but has a flattened trough and a mild implicit time shift. The single‐flash photopic ff‐ERG (LA 3) displays a normal a‐wave amplitude with a broadened trough; the waveform features a sharply rising b‐wave with no oscillatory potentials and a reduced b/a ratio. Variants in *NYX*, a three‐exon gene located on the X chromosome that codes for nyctalopin, represent the major gene defect responsible for cCSNB associated with high myopia (Zeitz et al., 2015). It is a leucine‐rich protein which is crucial for positioning the transient receptor potential cation channel subfamily M member 1 (*TRPM1*) at the dendritic tips of ON‐BCs (Xiao et al., 2006; Zhang et al., 2007).

**FIGURE 1:**
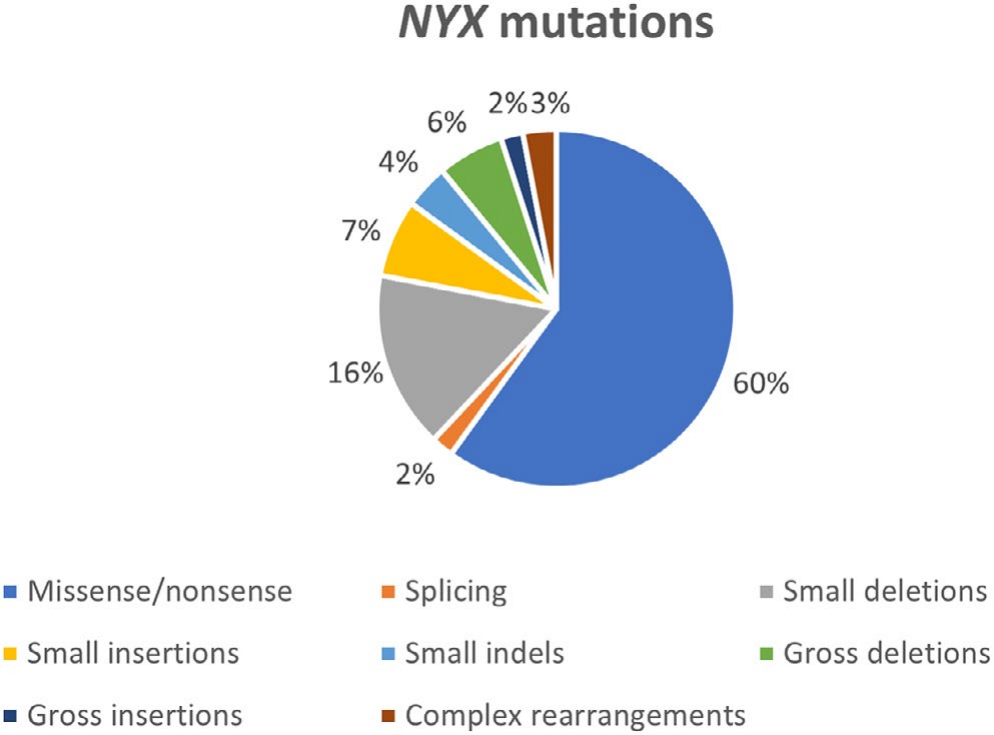
Mutation spectrum identified in *NYX*, with mutation types and their respective percentages among all discovered mutations.

There are more than 100 disease‐causing variants identified in *NYX* leading to cCSNB and myopia (Human Gene Mutation Database, 2025). These comprise mostly nonsense and missense variants, 65 in total. In addition, 29 small deletions/insertions/indels along with 9 frameshift insertions and deletions have been identified (Figure 1). Only two variants affecting splicing are known. Some variants in *NYX* were reported in association with isolated high myopia and presumably no functional defect suggesting cCSNB (Yip et al., 2013; Zhang et al., 2007; Zhou et al., 2015). However, no variants in *NYX* have been reported to cause cCSNB without high myopia.

Although several genes have been associated with cCSNB, there are still numerous cases where the origin of the condition is linked to unknown variants in known genes or novel gene defects to be discovered. These variants may be located in regions that are often overlooked in genetic testing, such as intronic or regulatory regions, since these variants were not previously considered as disease‐causing (Qian et al., 2021). The purpose of this study is to identify the genetic cause of an unresolved case of cCSNB.

## 2 MATERIALS AND METHODS

### 2.1 Ethics approval, clinical examination and DNA extraction

This is a historical case, clinically characterized about 10 years ago. The male subject underwent ophthalmic examination including best‐corrected visual acuity (BCVA), full‐field electroretinogram recording following the International Society for Clinical Electrophysiology of Vision (ISCEV) standard and multimodal retinal imaging as previously described (Marmor et al., 2009). Before genetic testing, informed consent was obtained from the participant. Research procedures adhered to the tenets of the Declaration of Helsinki and were approved by the local Ethics Committee. Blood samples of proband and family members were collected for genetic research and genomic DNA was extracted by standard methods.

### 2.2 Variant screening by Sanger, whole genome sequencing and bioinformatic analysis

**FIGURE 2:**
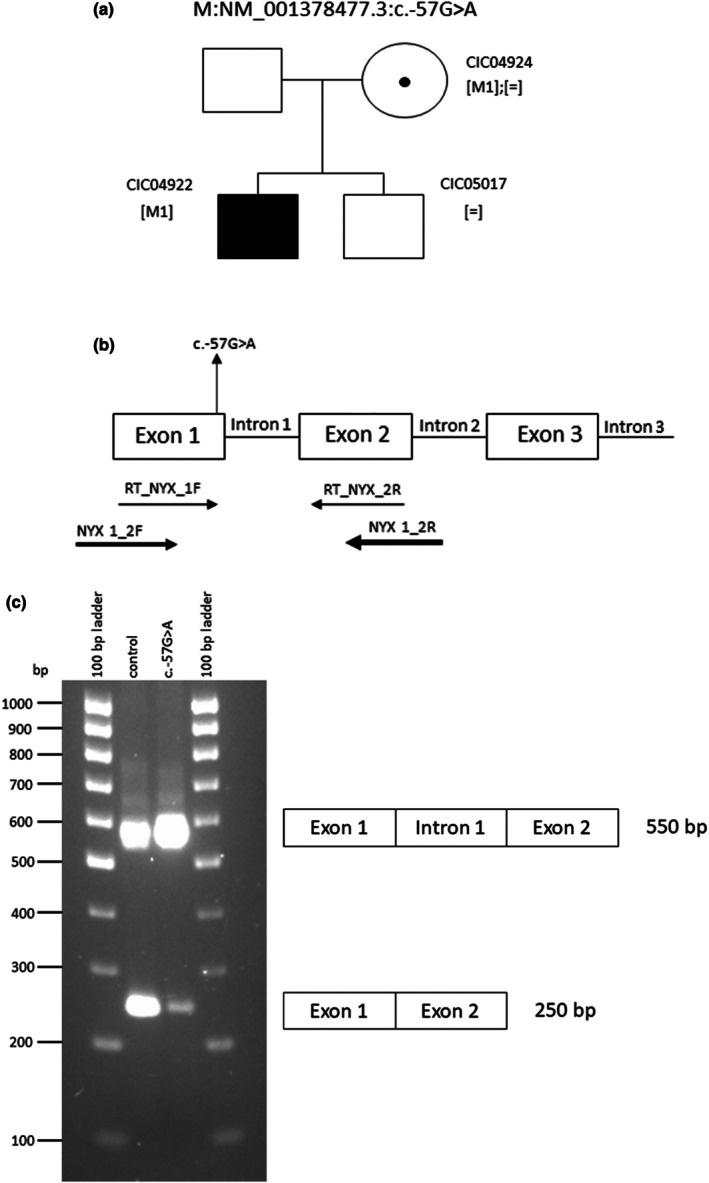
Minigene approach was used to validate the impact on splicing of the identified variant. (a) Pedigree of the family with one son affected by cCSNB, carrying a likely disease‐causing variant in the noncoding region of *NYX*. Square symbols: Males; round symbols: Females; filled symbols: Affected individuals; unfilled symbols: Unaffected individuals. (b) The genomic regions encompassing the variant and the control were amplified, showing genomic and exonic primers utilized for the minigene assays on both genomic and complementary DNA levels. (c) The visual presentation of an agarose gel run, featuring transcripts as samples.

Analysis of the coding regions of known genes underlying cCSNB was performed by Sanger sequencing. Detailed protocols will be delivered on request. Whole genome sequencing (WGS) of the proband and the unaffected mother was performed at a company IntegraGen (Evry, France) as previously described (Zeitz et al., 2024) (Figure 2a). WGS was done using NovaSeq 6000 with a coverage of 30X. Burrows‐Wheeler Aligner (BWA) tool was used to map the reads to the human genome build (hg38). SNV and small indel calling were performed using the Broad Institute's GATK Haplotype Caller GVCF tool (GATK 4.1.7.0). Variants were further annotated using Ensembl's VEP (Variant Effect Predictor, release VEP 95.1). Variants were prioritized based on a minor allele frequency (MAF) ≤ 0.0005 in the Genome Aggregation Database (gnomAD v4.1.0), focusing on insertions or deletions (InDels), nonsense, missense and splice site variants (GnomAD, 2025). Focus on the analysis of the WGS data was put on coding exons and flanking intronic regions of genes, implicated in CSNB: *NYX*, *VSX2* [MIM# 142993], *CABP4* [MIM# 608965], *CACNA2D4* [MIM# 608171], *GRM6*, *RHO* [MIM# 180380], *GNAT1* [MIM# 139330], *PDE6B* [MIM# 180072], *TRPM1*, *SLC24A1* [MIM# 603617], *GPR179*, *LRIT3*, *ELFN1* [MIM# 614964], *GNB3* [MIM# 139130], *RDH5* [MIM# 601617], *RBP4* [MIM# 180250] and *CNGB1* [MIM# 600724]. Their pathogenicity was assessed using bioinformatic tools such as SpliceAI, Pangolin, REVEL, CADD v1.7, BayesDel and MetaRNN, which leverage deleteriousness meta‐scores integrating multiple in silico predictors, allele frequency information and conservation scores (only for MetaRNN) (Ioannidis et al., 2016; Jaganathan et al., 2019; Li et al., 2022; Schubach et al., 2024; Tian et al., 2019; Zeng & Li, 2022). SpliceAI and Pangolin are programs that can predict whether a coding or noncoding variant creates, disrupts or alters the strength of a splice donor or splice acceptor site. They give a numeric value between 0 and 1, with a score closer to 1 indicating a high likelihood of pathogenicity, while a score closer to 0 suggesting benign variants (Zeng & Li, 2022). To assess the functional impact of noncoding variants, LINSIGHT (Gronau et al., 2013), ReMM (Schubach et al., 2024) and FunUV (Li et al., 2022) (specialized in UTR variants) were used. LINSIGHT scores >0.5 are considered indicative of regulatory disruption, while ReMM scores >0.8 and FunUV scores >0.9 are interpreted as high‐confidence predictions of pathogenic effects. To identify upstream open reading frames (uORFs) in the 5′UTR of the two *NYX* transcripts (NM_001378477.3 and NM_022567.3), uORFdb database was used. uORFdb provides a comprehensive annotation of uORFs based on experimental and computational evidence. The coordinates and sequences of the uORFs were extracted and analysed to assess their potential impact on translational regulation. We considered uORFs with a minimum length of 3 codons and an AUG start codon for further analysis.

### 2.3 Minigene approach

To test the pathogenic effect on splicing variant identified in the noncoding region of *NYX* (NG_009112.1: g.5057G>A, NM_001378477.3), a minigene approach was performed, as described before (Orhan et al., 2013; Zeitz et al., 2019). In order to target the variant located in exon 1 (c.‐57G>A, 5′ UTR region of mRNA), exons 1–2 were amplified on subject and control genomic DNA (Figure 2). Vector pBudCE4.1 (Thermo Fisher Scientific, Waltham, Massachusetts, USA) was linearised using *KpnI*‐HF enzyme (New England Biolabs, Ipswich, Massachusetts, USA), followed by the purification process that was done using a commercial kit (Macherey‐Nagel clean‐up kit, Düren, Nordrhein‐Westfalen, Germany). PCR fragments were cloned into a vector using a kit (NEBuilder® HiFi DNA Assembly Master Mix, New England Biolabs). The newly constructed vector was used to transform *E. coli* cells using a heat‐shock method (Addgene, 2025). Plasmids were isolated using a kit (Macherey‐Nagel NucleoSpin plasmid mini), and their concentrations were measured on a spectrophotometer (Nanodrop, Thermo Fisher Scientific). To assess the efficiency of the fusion reaction, isolated plasmids were digested with two restriction enzymes, *HindIII*‐HF and *NotI*‐HF (New England Biolabs). Samples were analysed on a 1% agarose gel and compared with molecular weight markers, 100 bp and 1 kb. To confirm the efficiency of the fusion reaction, Sanger sequencing was performed on the plasmids containing inserts. Samples were analysed on a sequencer machine (3730 DNA Analyser, Applied Biosystems, USA).

Following the successful procedure, mammalian COS‐1 cells were selected for transfection with the plasmids containing the insert of the control and mutant product using the CaCl_2_ transfection method (Sigma‐Aldrich, 2025). Cells were incubated until they reached confluence, after which they were harvested. RNA was then isolated using a kit (RNeasy Plus Mini Kit, Qiagen, Hilden, Germany). For semi‐quantitative RT‐PCR experiments, a kit (QuantiTect Reverse Transcription Kit, Qiagen) was used, with the following primers: RT *NYX*_1F: 5′ CCTGCACAAGTAACTATTCC 3′ and RT *NYX*_2R: 5′ CATGCAGAAGCAGGACCAAC 3′. RT‐PCR products were analysed on a 2% agarose gel and visualized with a gel documentation machine (Molecular Imager® Gel Doc™ XR+ System with Image Lab™ Software; Bio‐Rad, Life Science, Marnes‐la‐Coquette, France). Prominent bands were purified (NucleoSpinGel or PCR Clean‐up®, Macherey‐Nagel, Hoerdt, France) and cloned in a vector (Topo‐TA dual cloning vector, pCR™II‐TOPO®vector, Invitrogen, Thermo Fischer Scientific). These vectors were used to transform Top10 bacteria (Thermo Fisher Scientific) using the heat‐shock method. Plasmids were isolated using a kit (Macherey‐Nagel NucleoSpin plasmid mini) and Sanger sequenced to confirm the insertion. RT‐PCR experiments were repeated three times.

## 3 RESULTS

**FIGURE 3:**
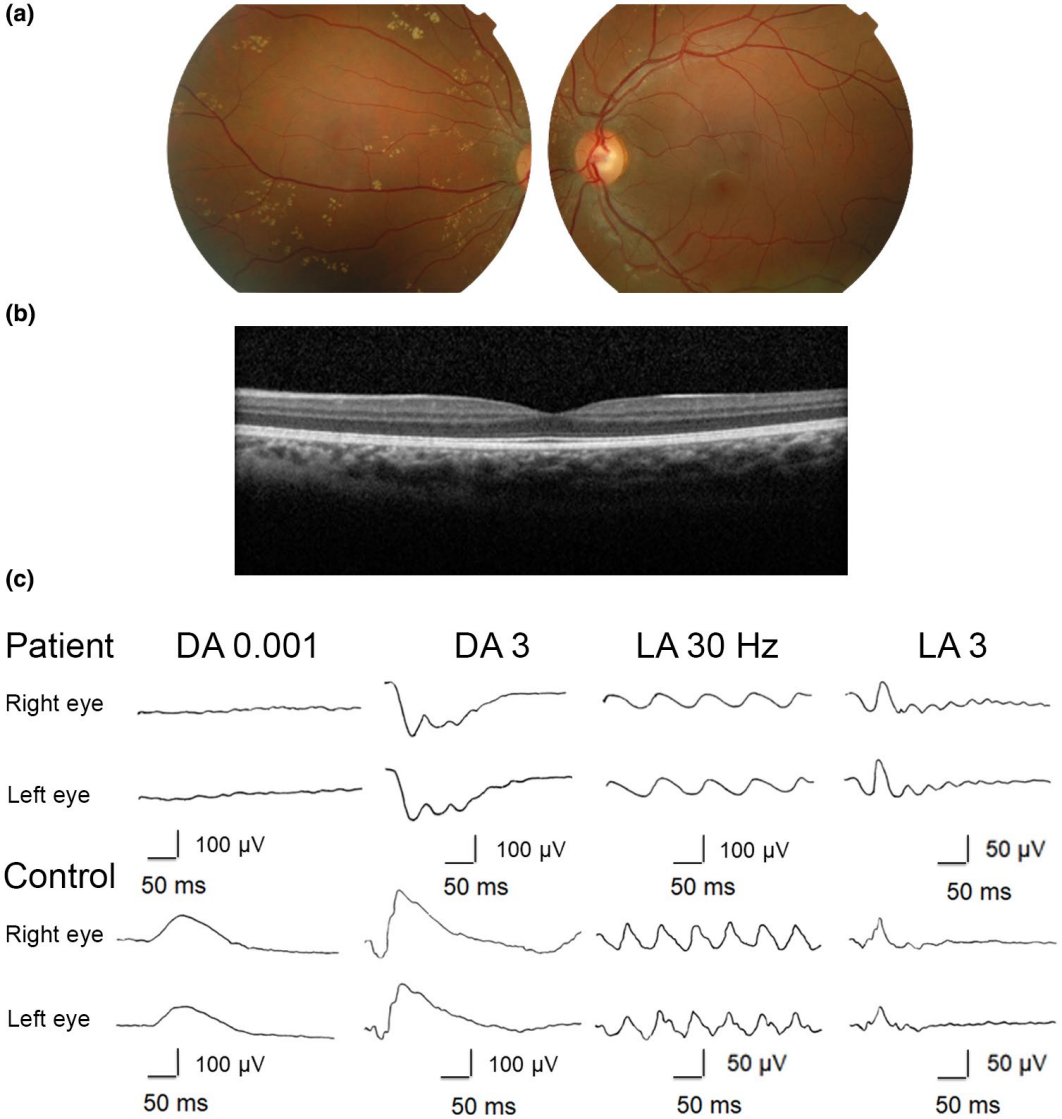
Retinal imaging and electrophysiological evaluation supported the diagnosis of congenital stationary night blindness. (a) Fundus imaging showed benign RPE depigmentation called ‘polar bear tracks’. (b) OCT showed normal retinal lamination. (c) ERG responses showed lack of b‐wave characteristic for cCSNB.

The 18‐year‐old male proband was referred to the ophthalmology clinic after the discovery of unilateral congenital albinotic spots of the retinal pigment epithelium (CASRPE), also known as polar bear tracks, in the left eye during a routine examination. Otherwise, the subject had an unremarkable medical and familial history. He was asymptomatic and had 20/20 visual acuity without any optical correction. The patient was essentially emmetropic, requiring a minimal correction of +0.25 D in both eyes. Axial length measurements were not performed, as this is not part of routine testing and the patient did not return for further evaluation. Ff‐ERG revealed, however, typical features of ON‐BC dysfunction: no b‐wave detected at the DA 0.01, a normal a‐wave but a severely reduced b‐wave at the DA 3 and DA 10, a square‐shaped a‐wave trough and a reduced b/a ratio at the LA 3 with reduced and delayed LA 30 Hz. Multimodal imaging was unremarkable beside the presence of polar bear track lesions on the left eye fundus (Figure 3). The patient was reassured on the fundus lesions, which are congenital and benign, but the diagnosis of cCSNB was made on ff‐ERG and the patient subsequently consented for genetic testing.

**FIGURE 4:**
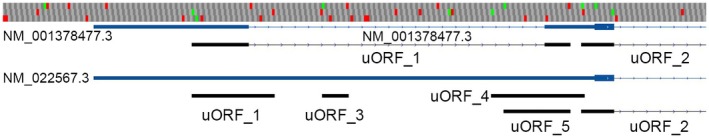
5′ UTRs of *NYX* transcripts with their uORFs. Both *NYX* transcripts are shown in blue and the uORFs in black. The three ORFs on the forward strand of the X chromosome are shown in grey, with the ATG codon in green and the stop codon in red.

Direct Sanger sequencing of the coding regions of genes underlying CSNB did not reveal any disease‐causing variants. These findings were confirmed by WGS. However, a deeper analysis of WGS using bioinformatic prediction tools identified a putative causal variant in *NYX* (NG_009112.1: g.5057G>A, NM_001378477.3: c.‐57G>A). This variant is located in the noncoding region of the gene (c.‐57G>A) and specifically affects the last nucleotide of exon 1 in the 5′ UTR region of *NYX* (Figure 2b). It is predicted to lead to the loss of the donor site, with a delta score of 0.19 according to SpliceAI and 0.64 according to Pangolin. LINSIGHT assigned a score of 0.21, suggesting that the variant is unlikely to disrupt regulatory elements such as enhancers or promoters. By contrast, ReMM predicted a high likelihood of the variant being a pathogenic regulatory mutation, with a score of 0.90. Finally, FunUV assigned a score of 0.99, indicating a strong predicted disruption of 5′ UTR stability and/or translation efficiency. These predictions indicate that the variant is most likely pathogenic primarily due to its moderate impact on splicing and a strong predicted disruption of translation efficiency. Sanger sequencing confirmed that the variant segregated with the phenotype in the family (Figure 2a). Bioinformatic analysis with a minigene assay validated the pathogenic character of this novel variant in the noncoding region of *NYX*. Indeed, semi‐quantitative RT‐PCR experiments revealed a different transcript profile in the mutant setting compared with the control (Figure 2c). Although both the control and the mutant minigene constructs produce a low to moderate impact on transcript, encompassing exon 1, the former intron 1 (Ex1’, Figure 2b), and exon 2, which represent the transcript NM022567.3, or exon 1 and exon 2, which represents the transcript NM_001378477.3. The c.‐57G>A variant seems to lead to a lower amount of the lower molecular weight exon 1 exon 2 transcript (NM_001378477.3) but does not affect the coding region of *NYX*, which starts in exon 2 (Figure 2b). Therefore, this variant may influence the expression of nyctalopin but most likely does not lead to a complete loss of nyctalopin as found with other cCSNB‐related *NYX* variants. These findings were confirmed by Sanger sequencing. Thus, our findings indicate that the c.‐57G>A variant disrupts the splicing process by causing incomplete excision of intron 1, leading to a reduced quantity of a low molecular weight transcript that contributes to cCSNB but lacking high myopia. Both transcripts contain several small open reading frames (uORFs), which are located upstream of the main coding sequence, usually within the 5′ untranslated region (5′‐UTR) (Figure 4).

uORF_2 is an N‐terminal extension uORF and is present in both transcript (Figure 4), although its Kozak sequence context is weak (Table 1), suggesting a lower efficiency of translation initiation. uORF_1, uORF_3, uORF_4 and uORF_5 are non‐overlapping uORFs. uORF_1 share the same start codon in both transcripts but has a different stop due to the presence of the intron. Notably, uORF_1 exhibits a strong Kozak sequence context, indicating a high potential for translation initiation. uORF_3 and uORF_5 have an adequate Kozak sequence context, while uORF_4 also exhibits a strong Kozak sequence context. uORF_3, uORF_4 and uORF_5 are exclusively present in the NM_022567.3 transcript with their start codons located within the intron.

**TABLE 1:** Translation efficiency is strong in uORF_1 and uORF_4.

uORF	Kozak sequence context	Kozak sequence strength
uORF_1	TGGGAGATG G	Strong
uORF_2	GGGTGGATGA	Weak
uORF_3	TCAGTGATGC	Adequate
uORF_4	GGAGGCATG G	Strong
uORF_5	GTTCTCATG G	Adequate

## 4 DISCUSSION

Around 30% of subjects with IRD lack disease‐causing variants after sequencing of coding and flanking exonic regions of known genes. To identify the variants underlying the condition, sequencing methods combined with bioinformatics tools and functional analyses are being employed. Among IRDs, cCSNB is characterized by a very specific functional signature on the electroretinogram in keeping with ON‐BC dysfunction, with subjects usually experiencing non‐progressive night blindness and other ocular signs such as high myopia. cCSNB is caused by variants in several genes, most commonly in *NYX*, followed by *TRPM1*, *GRM6*, *GPR179* and *LRIT3* (Zeitz et al., 2015). These variants are usually identified in coding regions using Sanger sequencing and targeted next generation sequencing, whole exome sequencing or WGS, focusing on the coding parts of the genome (Miyake, 1986; Miyake, 1989; Masu et al., 1995; Pusch et al., 2000; Bech‐Hansen et al., 2000; Gregg et al., 2003; Dryja et al., 2005; Zeitz et al., 2005; Zeitz et al., 2005; Zeitz, 2007; MacDonald et al., 2008; Audo et al., 2009; van Genderen et al., 2009; Audo et al., 2012; Peachey et al., 2012; Neuillé et al., 2014; Zeitz et al., 2015; Huang et al., 2024).

Today, more than 100 disease‐causing *NYX* variants have been identified, which lead to cCSNB and other ocular signs such as high myopia (HGMD, 2025). They comprise missense and truncating variants all predicted to lead to loss of function (Figure 1). While the localization of the respective mutant nyctalopin protein seems not to be affected (Zeitz et al., 2003), it has been shown that nyctalopin is important together with other proteins, such as LRIT3, to correctly localize TRPM1 at the cell membrane (Neuillé et al., 2015; Pearring et al., 2011). Here, we identified a case with a specific ERG signature of cCSNB, harbouring a 5′ UTR variant in *NYX* (c.‐57G>A), predicted to affect a donor site leading to intron 1 retention, lower exon 1 exon 2 expression and most likely lower nyctalopin protein amount but not to loss of function of nyctalopin.

Interestingly, this reported case is atypical: It manifests the specific ON‐BC functional defect associated with cCSNB but does not display the other clinical signs usually linked to the disease, such as myopia, nystagmus, decreased vision and night blindness. The latter symptom may be overlooked, especially when living in a major city with bright night lighting, as it was the case for this patient. Nevertheless, the identification of a 5′UTR variant that causes downregulation of *NYX* expression of NM_001378477.3 and most likely reduction of nyctalopin protein may suggest that even a small amount of normal protein may be sufficient for normal visual acuity and myopia prevention but is not enough to fully maintain signal transduction.

Although the *NYX* variant does not change the presence of the distinct uORFs compositions and Kozak sequence of both transcripts, the lower amount of NM_001378477.3 will also alter the composition. uORF_2, present in both transcripts, is unlikely to contribute to any potential differences in protein levels, as it has a weak Kozak sequence context, reducing its efficiency in initiating translation. Instead, the key differences arise from four additional uORFs (uORF_1, uORF_3, uORF_4 and uORF_5) that are either modified or exclusively present in NM_022567.3. uORF_1 shares the same start codon in both transcripts but has a different stop codon in NM_022567.3 due to intron inclusion. Its strong Kozak sequence context suggests it could efficiently sequester ribosomes, potentially reducing translation of the main *NYX* coding sequence (CDS). uORF_3 and uORF_5, exclusive to NM_022567.3, have adequate Kozak sequence contexts, allowing them to initiate translation and further divert ribosomes. uORF_4, also exclusive to NM_022567.3, has a strong Kozak sequence context, enhancing its potential to inhibit main *NYX* CDS translation. Thus, we propose that these additional uORFs, particularly those with strong Kozak contexts (uORF_1 and uORF_4), could reduce ribosome availability for the main *NYX* CDS in NM_022567.3, potentially leading to lower protein production compared with NM_001378477.3. Further experimental validation, such as ribosome profiling or reporter assays, would be necessary to confirm this hypothesis.

**FIGURE 5:**
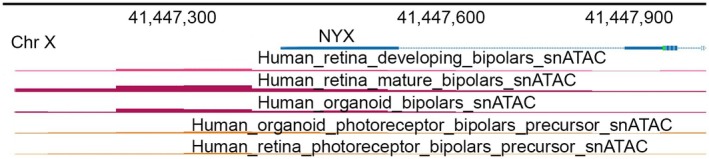
snATAC‐seq signals in the 5′ UTR of the *NYX* gene. The *NYX* gene is shown in blue, while snATAC‐seq signals in bipolar cells and photoreceptor–bipolar precursor cells are shown in pink and orange, respectively.

In addition, the *NYX* variant identified herein is located within a chromatin‐accessible region (Figure 5), as identified by single‐nucleus ATAC‐seq (snATAC‐seq) data specifically in bipolar cells (Thomas et al., 2022). Chromatin accessibility in this region suggests active regulatory potential, which may influence both splicing and translational regulation. These findings reinforce our hypothesis that this variant may lead to this bipolar cell‐specific cCSNB phenotype, albeit the ubiquitous expression of *NYX*. This is in accordance with our minigene approach revealing a lower quantity of the NYX transcript NM_001378477.3, which is predicted to produce under normal conditions functional nyctalopin.

By contrast, other pathogenic *NYX* variants reported so far were predicted to cause a complete loss of protein function, resulting in a fully penetrant phenotype, including cCSNB and high myopia. A recent study from our laboratory highlighted differentially expressed genes and pathways in cCSNB which may explain high myopia in cCSNB (Zeitz et al., 2023). Our findings could suggest that this noncoding variant in *NYX* may lead to mislocalization of TRPM1, while the expression levels of genes important for development or protection of myopia in the retina are maintained. Further functional assays are needed to investigate the influence of this noncoding *NYX* variant identified herein in comparison with published loss of function variants showing a full penetrant phenotype, for example, by performing luciferase studies or in vivo modelling, using mouse models to study the potential effects of the variants on the regulation of *NYX* expression or on the stability and translation efficiency of its transcripts.

Our findings are in accordance with previously published studies. Indeed, more recently, variants in noncoding regions have been found to be disease‐causing in genes associated with various IRDs, such as CSNB (Di Scipio et al., 2020; Zeitz et al., 2019), Usher syndrome (Khan et al., 2017; Liquori et al., 2016), Leber congenital amaurosis (Coppieters et al., 2015; den Hollander et al., 2006) or Stargardt disease (Braun et al., 2013), indicating the relevance to screen these regions in cases with IRD lacking variants in coding regions of known gene defects. These variants may affect gene expression regulators such as enhancers, silencers and binding sites for transcription factors. They often involve dysregulation of gene‐splicing mechanisms, which lead to unstable mRNA transcripts or their degradation. This may be accompanied by exon skipping or intron retention. Consequently, at the protein level, this results in reduced protein translation (Gaildrat et al., 2010), as also predicted in our case.

This case highlights the importance of identifying pathognomonic phenotypic signatures, such as those on ERG recordings, to guide genetic investigations that will enable a comprehensive approach, including genome sequencing, bioinformatic analysis focusing on known gene defects and in vitro validations. Hunting for novel gene defects should start only after exclusion of disease‐causing variants in all genomic regions and chromosomal rearrangements of known genes. Indeed, to the best of our knowledge, this is the first documented case of a noncoding variant in *NYX* associated with CSNB in the absence of high myopia. These findings underscore the clinical relevance of investigating noncoding regions in known IRD genes, particularly in cases that remain genetically unexplained. Further research is needed to determine whether the development of high myopia in cCSNB is influenced by the type and location of *NYX* variants. Together, these approaches are expected to soon uncover the genetic basis of the remaining 30%–50% of unresolved IRD cases.

## FUNDING INFORMATION

This research was funded by Fondation Voir et Entendre (C.Z.); French state funds managed by the Agence Nationale de la Recherche within the Investissements d'Avenir program (ANR‐11‐IDEX‐0004‐0); IHU FOReSIGHT (ANR‐18‐IAHU‐0001 to I.A. and C.Z.); ANR‐23‐CE17‐0014‐01 RP_SOLVEANDCURE (to C.Z. and I.A.); LABEX LIFESENSES (ANR‐10‐LABX‐65 to I.A. and C.Z.); Retina France (I.A. and C.Z.); Foundation Fighting Blindness Center grant (CCMM‐0907‐0428‐INSERM04 to I.A. and C.Z.); and grant BR‐GE‐0619‐0761‐INSERM (to I.A. and C.Z.). This work was supported by the Marie Skłodowska‐Curie Actions (MSCA) under the European Union's Horizon [101119501—MyoTreat] research and innovation programme (F.S). Funded by the European Union (under GA # 101119501). Views and opinions expressed are, however, those of the author(s) only and do not necessarily reflect those of the European Union or European Research Executive Agency. Neither the European Union nor the granting authority can be held responsible for them.
